# Evaluation of GPS/BDS-3 PPP-AR Using the FCBs Predicted by GA-BPNN Method with iGMAS Products

**DOI:** 10.3390/s25226952

**Published:** 2025-11-13

**Authors:** Jin Wang, Guangyao Yang, Qiong Liu, Ying Xu

**Affiliations:** 1 College of Geodesy and Geomatics, Shandong University of Science and Technology, Qingdao 266590, China; wangjin@sdust.edu.cn (J.W.); 202382020082@sdust.edu.cn (G.Y.); yingxu@sdust.edu.cn (Y.X.); 2 Chinese Academy of Surveying and Mapping, Beijing 100830, China; 3 Hainan Province Hydrogeological Engineering Geological Survey Institute Co., Ltd., Haikou 571100, China

**Keywords:** GPS/BDS-3, PPP-AR, FCB prediction, GA-BPNN, iGMAS

## Abstract

**Highlights:**

**What are the main findings?**

- A Genetic Algorithm Optimized Backpropagation Neural Network model is developed to predict Wide-Lane and Narrow-Lane Fractional Cycle Bias products for GPS and BDS-3.

**What is the implication of the main finding?**

- The FCB prediction model provides an efficient and reliable approach for real-time FCB acquisition, effectively reducing computational complexity and improving efficiency.
- The PPP-AR performance using the predicted FCB matches that of official iGMAS products, confirming the feasibility and practicality of GA-BPNN-based FCB prediction in precise positioning applications.

**Abstract:**

Ambiguity Resolution (AR) is regarded as an effective technique for enhancing positioning accuracy and reducing convergence time in Precise Point Positioning (PPP). However, the Wide-Lane Fractional Cycle Bias (WL FCB) and Narrow-Lane Fractional Cycle Bias (NL FCB) needed for AR are generated from network solutions based on numerous globally distributed stations, leading to considerable computational load and processing time. A prediction model for FCB is proposed using the Genetic Algorithm Optimized Backpropagation Neural Network (GA-BPNN), and high-precision predictions of WL and NL FCB for Day of Year (DOY) 321 in 2023 are successfully achieved. Comparisons with iGMAS products show that predicted WL FCB deviations are within 0.01 cycles, and predicted NL FCB over 12 h deviates within 0.1 cycles (excluding satellite C20). The performance of three PPP schemes, Float, Fixed (based on FCB from iGMAS), and BP-Fixed (based on FCB predicted by GA-BPNN), is compared through experiments. For GPS + BDS-3, the accuracies of the BP-Fixed scheme are 0.0034 m, 0.0039 m, and 0.0100 m in the east, north, and up directions, respectively. The ambiguity fixed rates reach 98.62% for BP-Fixed. These outcomes confirm that the positioning performance using the predicted FCB of GA-BPNN is highly consistent with that using FCB products.

## 1. Introduction

Global Navigation Satellite Systems (GNSS) can deliver high-precision positioning, navigation, and timing services to users worldwide by utilizing signals transmitted by satellites [1]. The primary GNSS offering global navigation and positioning services include Global Positioning System (GPS), BeiDou Navigation Satellite System (BDS), Galileo Satellite Navigation System (Galileo), and Global Navigation Satellite System (GLONASS) [2]. As a significant technique of GNSS positioning, Precise Point Positioning (PPP) can be performed using only a single receiver to obtain high-precision point coordinates. It is distinguished by its strong applicability, high flexibility, and minimal operational requirements [3].

Currently, the main factor limiting the further development and application of PPP is excessively long convergence time [4]. Precise Point Positioning-Ambiguity Resolution (PPP-AR) is one of the key technologies ensuring that PPP can rapidly provide stable and reliable high-precision positioning information [5]. Fractional Cycle Bias (FCB) method was proposed by Ge et al. (2008), in which uncalibrated phase delays (UPD) of satellite-side single difference were calculated using observation data from approximately 180 GPS stations globally, thereby enabling PPP-AR [6]. Experimental results showed that over 80% of ambiguities could be fixed, with improvements in both convergence time and positioning accuracy. Furthermore, the Integer Recovery Clock (IRC) method was proposed to achieve PPP-AR by utilizing FCB and integer clock products. Experiments indicated that the ambiguity resolution success rate reached 88.5% at selected IGS stations, with significant improvement in PPP accuracy, achieving a level of 2–3 cm [7]. The decoupled clock model (DCM) was proposed, in which the clock errors for pseudorange and carrier-phase observations were decoupled, and the code clock and phase clock were estimated separately to achieve PPP-AR [8]. These three methods were shown to be theoretically equivalent, despite differences in their algorithmic implementations [9]. The observable-specific signal bias (OSB) method was developed by applying the S-system theory to resolve the rank deficiency inherent in the undifferenced and uncombined observation model, thus significantly promoting the practical implementation of PPP-AR [10].

The implementation of PPP-AR using the FCB method is dependent on corresponding Wide-Lane Fractional Cycle Bias (WL FCB) and Narrow-Lane Fractional Cycle Bias (NL FCB) products [11,12]. The rank deficiency problem in the model was effectively addressed through the introduction of a reference receiver FCB within a global network of satellites and stations. Subsequently, satellite WL FCB and NL FCB products were generated through least-squares estimation [13]. Since 2015, WLFCB and NLFCB products for GPS satellites have been made available by the School of Geodesy and Geomatics (SGG) at Wuhan University [14]. Similarly, beginning in 2021, multi-GNSS WLFCB and NLFCB products, including those for BDS-3 satellites, have been released by the International GNSS Monitoring and Assessment System (iGMAS) [15]. The performance and applicability of PPP-AR are directly influenced by the quality and accessibility of these FCB products. Users can directly employ these publicly available FCB corrections to achieve PPP-AR across different satellite systems [16]. Furthermore, related research and development have been significantly advanced in recent years by the emergence of specialized FCB estimation software. An open-source software package named GREAT-UPD was developed for estimating Extra-Wide-Lane (EWL), WL, and NL FCB for GPS, GLONASS, Galileo, and BDS-2 constellations, accompanied by integrated preprocessing and analysis utilities. The generated FCB products were characterized by high stability and reliability, effectively supporting the implementation of multi-frequency and multi-system PPP-AR [17]. Another open-source tool, M_FCB, was developed based on MATLAB, specializing in FCB estimation including BDS-3 satellites [18]. These open-source tools provided alternative channels for acquiring FCB products, while their transparent code architecture facilitated a thorough understanding of FCB estimation methodologies and enabled subsequent development.

With the growing demand for real-time navigation, higher requirements are imposed on the immediacy of positioning results [19]. To achieve AR in Real-Time Precise Point Positioning (RT-PPP), the estimation of real-time FCB is considered particularly critical [20]. However, significant challenges are encountered in acquiring real-time FCB [21]: traditional FCB estimation methods require the processing of massive observation data and the execution of complex adjustment calculations, which are excessively time-consuming and hardly meet the demand for second-level updates in RT-PPP. Meanwhile, longer delays are inevitably introduced in the products provided by analysis centers, from data collection, processing, and quality control to final distribution to users. Therefore, there is an urgent need to explore novel approaches for FCB acquisition that can circumvent complex global computations and substantially reduce product generation time. It is under this background that Artificial Neural Network (ANN) have emerged, offering a new perspective to address this challenge.

With the rapid progress in artificial intelligence, ANN, particularly the Backpropagation Neural Network (BPNN), have found extensive use in the GNSS field owing to their exceptional nonlinear mapping capability and pattern recognition characteristics [22], demonstrating unique advantages in GNSS data modeling, error correction, and accuracy enhancement [23]. A tropospheric delay phase spatial prediction method based on BPNN and Zenith Total Delay (ZTD) was proposed, which effectively captured the complex relationship between discrete site data and topographic elevation, significantly improving the accuracy of ZTD spatial interpolation [24]. Modeling of the orbit error variations in BDS-2/GPS broadcast ephemeris was conducted using BPNN and other models to enhance the precision of the broadcast ephemeris [25]. The global search capability of BPNN was enhanced, achieving a prediction accuracy of 0.064 ns for the one-hour satellite clock bias (SCB) of BDS-3 satellites and realizing centimeter-level static PPP accuracy [26]. The BPNN, combined with the modified gravity–geologic method (MGGM), was employed to develop a regional model for the South China Sea, and the reliability of BPNN in modeling complex geospatial nonlinear relationships was further validated [27]. In addition, a positioning error prediction model based on BPNN was constructed, which effectively suppressed the divergence of position errors during GPS outages and provided a reliable solution for scenarios involving GNSS signal interruptions [28].

The training of BPNN is a complex and time-consuming process [29]. Essentially, error information is propagated back to various parameters through gradient descent, while the solution space is traversed for optimizing the thresholds and weights [30]. The Genetic Algorithm (GA) continuously optimizes the gene combinations of individuals by simulating natural selection, crossover, and mutation, thereby gradually approaching the optimal solution [31]. In the Genetic Algorithm Optimized Backpropagation Neural Network (GA-BPNN) model, mutation, crossover, and selection operations are used to replace the gradient descent method in BPNN, which effectively enhances the training speed of the neural network and reduces convergence time [32]. The GA-BPNN was applied by Niu et al. (2025) to predict polar motion (PM) [33]. The results demonstrated that the Mean Absolute Error (MAE) of predictions generated by the GA-BPNN model on the 1st, 50th, and 200th days was significantly lower than that of the BPNN model. Sun et al. (2024) addressed the issue of long-term or intermittent GNSS signal blockage in complex environments by utilizing GA-BP to predict IMU position corrections [34]. The weights between the GNSS float solutions and the GA-BP predicted values were adaptively adjusted, which effectively suppressed the divergence of positioning errors. Huang et al. (2025) established a dynamic positioning error correction model using raw GNSS observations, position, and velocity information via GA-BPNN, significantly improving the positioning precision of smartphones in complex environments [35].

In this study, a modeling and prediction algorithm for FCB based on GA-BPNN is proposed. After constructing continuous and stable FCB time series for GPS and BDS-3 satellites, an autoregressive training dataset is established using the sliding window method to achieve high-accuracy prediction of both WL and NL FCB products. The main contributions of this study are as follows: (1) The constructed FCB prediction model significantly enhances the accuracy and efficiency of real-time FCB acquisition. It provides a novel perspective and an effective solution for the acquisition of FCB, while enabling the precise realization of PPP-AR. (2) A detailed evaluation of the stability and accuracy of FCB products provided by the iGMAS is conducted, confirming their suitability for the GA-BPNN prediction model. (3) The performance of the predicted FCB products is validated, demonstrating that their positioning accuracy and ambiguity resolution rates are comparable to those of the iGMAS FCB products.

## 2. Theory and Methodology

In this section, the overall procedure of PPP-AR is first briefly outlined. Then, the fundamental theories and modeling approaches of the BPNN and GA-BPNN models are introduced. Finally, the metrics used for evaluating the performance of the predicted FCB products are proposed.

### 2.1. PPP Method

#### 2.1.1. Ionosphere-Free Combination Model

In PPP, the positioning is primarily performed by utilizing pseudorange and carrier-phase observations [36]. The measurement equations can be expressed as

(1) $$\left\{\begin{array}{l} P_{i}=\rho +c(dt_{r}-dt^{s})+T+I_{i}+d_{r,i}-d_{i}^{s}+\varepsilon_{r,i}^{s}\\ L_{i}=\rho +c(dt_{r}-dt^{s})+T-I_{i}+\lambda_{i}(N_{i}+b_{r,i}-b_{i}^{s})+\xi_{r,i}^{s} \end{array}\right.$$

where i denotes the frequency band; c denotes the speed of light in vacuum; r and s denote the receiver and satellite, respectively; ρ stands for the distance between the receiver and the satellite; $P_{i}$ and $L_{i}$ refer to the pseudorange and carrier-phase observations, respectively; $I_{i}$ denotes the ionospheric delay; $dt_{r}$ represents the receiver clock biases; $dt^{s}$ represents the satellite clock biases; T denotes the tropospheric delay; $\lambda_{i}$ denotes the carrier wavelength; $N_{i}$ denotes the integer ambiguity; $d_{r,i}$ is the Uncalibrated Code Delays (UCD) at the receiver; $d_{i}^{s}$ is the UCD at the satellite ends; $b_{r,i}$ is the UPD for the receiver; $b_{i}^{s}$ is the UPD for the satellite; and $\varepsilon_{r,i}^{s}$ and $\xi_{r,i}^{s}$ represent the residuals in pseudorange and carrier-phase observations, respectively.

To address the ionospheric delay parameter in Equation (1), an ionosphere-free combination model is typically formed through a combination of double frequency measurements [37], which can be expressed as

(2) $$\left\{\begin{array}{cc} P_{IF} & =\frac{f_{i}^{2}}{f_{i}^{2}-f_{j}^{2}}\cdot P_{i}-\frac{f_{j}^{2}}{f_{i}^{2}-f_{j}^{2}}\cdot P_{j}\\ & =\rho +c(dt_{r}-dt^{s})+T+d_{r,IF}-d_{IF}^{s}+\varepsilon_{r,IF}^{s}\\ L_{IF} & =\frac{f_{i}^{2}}{f_{i}^{2}-f_{j}^{2}}\cdot L_{i}-\frac{f_{j}^{2}}{f_{i}^{2}-f_{j}^{2}}\cdot L_{j}\\ & =\rho +c(dt_{r}-dt^{s})+T+\lambda_{IF}(N_{IF}+b_{r,IF}-b_{IF}^{s})+\xi_{r,IF}^{s} \end{array}\right.$$

where

(3) $$\left\{\begin{array}{l} d_{r,IF}=\frac{f_{i}^{2}}{f_{i}^{2}-f_{j}^{2}}\cdot d_{r,i}-\frac{f_{j}^{2}}{f_{i}^{2}-f_{j}^{2}}\cdot d_{r,j}\\ d_{IF}^{s}=\frac{f_{i}^{2}}{f_{i}^{2}-f_{j}^{2}}\cdot d_{i}^{s}-\frac{f_{j}^{2}}{f_{i}^{2}-f_{j}^{2}}\cdot d_{j}^{s}\\ N_{IF}=\frac{c\cdot (f_{i}N_{r,i}-f_{j}N_{r,j})}{\lambda_{IF}(f_{i}^{2}-f_{j}^{2})}\\ b_{r,IF}=\frac{c\cdot (f_{i}b_{r,i}-f_{j}b_{r,j})}{\lambda_{IF}(f_{i}^{2}-f_{j}^{2})}\\ b_{IF}^{s}=\frac{c\cdot (f_{i}b_{i}^{s}-f_{j}b_{j}^{s})}{\lambda_{IF}(f_{i}^{2}-f_{j}^{2})} \end{array}\right.$$

i and j represent different frequency bands; $P_{IF}$ and $L_{IF}$ denote the ionosphere-free combined pseudorange and carrier-phase observations, respectively; $f_{i}$ and $f_{j}$ represent the frequencies of different signals; c denotes the speed of light; $N_{r,IF}$ denotes the ionosphere-free ambiguity parameter; $d_{r,IF}$ and $d_{IF}^{s}$ represent the ionosphere-free UCD at the receiver and satellite, respectively; and $b_{r,IF}$ and $b_{IF}^{s}$ represent the ionosphere-free UPD at the receiver and satellite, respectively.

#### 2.1.2. Ambiguity Resolution Model

In Equation (1), the ambiguities of the carrier frequencies i and j are not readily and directly solved. The WL/NL combinations are introduced to map carrier-phase ambiguities into forms that facilitate integer ambiguity resolution and the fixing of the ionosphere-free linear-combination ambiguity.

(1) For any given epoch, the WL ambiguity of a specific satellite can be directly calculated using the Melbourne–Wübbena (MW) combination [38], which is expressed as

(4) $$\lambda_{WL}{\tilde{N}}_{WL}=\frac{f_{i}L_{i}-f_{j}L_{j}}{f_{i}-f_{j}}-\frac{f_{i}P_{i}+f_{j}P_{j}}{f_{i}+f_{j}}$$

where $\lambda_{WL}$ denotes the wavelength of the WL ambiguity, and ${\tilde{N}}_{WL}$ represents the float WL ambiguity.

The ${\tilde{N}}_{WL}$ estimated from the MW combination is influenced by errors and noise. To reduce these errors, an averaging method over multiple epochs is generally applied, and the smoothed WL ambiguity can then be expressed as

(5) $${\hat{N}}_{WL}=\langle {\tilde{N}}_{WL}\rangle$$

where 〈·〉 denotes averaging over multiple epochs, and ${\hat{N}}_{WL}$ represents the smoothed float WL ambiguity.

Since the FCB of the receiver end is common for different satellites, the single difference between satellites can be employed to eliminate FCB at the receiver end. Meanwhile, the FCB at the satellite end can be corrected using WL FCB products. The single-difference WL ambiguity after FCB correction is expressed as

(6) $${\bar{N}}_{WL}^{s_{k},s_{0}}=({\hat{N}}_{WL}^{s_{k}}-{\hat{N}}_{WL}^{s_{0}})-(FCB_{WL}^{s_{k}}-FCB_{WL}^{s_{0}})$$

where $s_{k}$ and $s_{0}$ represent the *k*-th satellite and the reference satellite, respectively; $FCB_{WL}^{s_{k}}$ and $FCB_{WL}^{s_{0}}$ represent the WL FCB products of the corresponding satellites.

Due to the long wavelength of WL ambiguity, the single-difference WL fixed solution can be obtained by rounding ${\bar{N}}_{WL}^{s_{k},s_{0}}$.

Subsequently, the single-difference NL float ambiguity can be calculated using the ionosphere-free combination model and the single-difference WL fixed solution, which is expressed as

(7) $${\hat{N}}_{NL}^{s_{k},s_{0}}=\frac{f_{i}+f_{j}}{C}\cdot \lambda_{IF}{\hat{N}}_{IF}^{s_{k},s_{0}}-\frac{f_{j}}{f_{i}-f_{j}}\cdot {\bar{N}}_{WL}^{s_{k},s_{0}}$$

where ${\hat{N}}_{NL}^{s_{k},s_{0}}$ denotes the single-difference NL float ambiguity, and ${\hat{N}}_{IF}^{s_{k},s_{0}}$ represents the single-difference ionosphere-free float ambiguity.

The NL FCB products are applied to correct the ambiguity, restoring the integer characteristics of the single-difference NL ambiguity:

(8) $${\bar{N}}_{NL}^{s_{k},s_{0}}={\hat{N}}_{NL}^{s_{k},s_{0}}-(FCB_{NL}^{s_{k}}-FCB_{NL}^{s_{0}})$$

where ${\bar{N}}_{NL}^{s_{k},s_{0}}$ denotes the corrected single-difference NL ambiguity; $FCB_{NL}^{s_{k}}$ and $FCB_{NL}^{s_{0}}$ represent the NL FCB products of the corresponding satellites.

Because the wavelength of the NL ambiguity is relatively short, it cannot be fixed directly by rounding. Therefore, the Least-squares AMBiguity Decorrelation Adjustment (LAMBDA) is typically applied to fix the single-difference NL ambiguity [39].

Finally, the single-difference ionosphere-free ambiguity with integer characteristics can be recovered using the single-difference NL fixed solution and the single-difference WL fixed solution, which is expressed as

(9) $$\lambda_{IF}{\bar{N}}_{IF}^{s_{k},s_{0}}=\frac{f_{j}}{f_{i}^{2}-f_{j}^{2}}\cdot c{\bar{N}}_{WL}^{s_{k},s_{0}}+\lambda_{NL}\cdot {\bar{N}}_{NL}^{s_{k},s_{0}}$$

where ${\bar{N}}_{IF}^{s_{k},s_{0}}$ denotes the single-difference ionosphere-free ambiguity with integer characteristics, $\lambda_{NL}$ denotes the wavelength of the NL ambiguity, and c denotes the speed of light in vacuum.

(2) ${\tilde{N}}_{WL}$ in Equation (4) can be re-expressed as follows:

(10) $$\left\{\begin{array}{cc} {\tilde{N}}_{WL} & =(\frac{f_{i}L_{i}-f_{j}L_{j}}{f_{i}-f_{j}}-\frac{f_{i}P_{i}+f_{j}P_{j}}{f_{i}+f_{j}})/\lambda_{WL}\\ & =N_{WL}+B_{r,WL}-B_{WL}^{s}\\ B_{WL}^{s} & =b_{i}^{s}-b_{j}^{s}-\frac{d_{IF}^{s}}{\lambda_{WL}}\\ B_{r,WL} & =b_{r,i}-b_{r,j}-\frac{\lambda_{NL}}{\lambda_{WL}}(\frac{d_{r,i}}{\lambda_{i}}+\frac{d_{r,j}}{\lambda_{j}}) \end{array}\right.$$

where $N_{WL}$ denotes the integer WL ambiguity, and $B_{r,WL}$ and $B_{WL}^{s}$ represent the receiver-end and satellite-end WL FCB, respectively.

Similarly, ${\hat{N}}_{NL}$ in Equation (7) can be re-expressed as follows:

(11) $$\left\{\begin{array}{cc} {\hat{N}}_{NL} & =\frac{f_{i}+f_{j}}{c}\cdot \lambda_{IF}{\hat{N}}_{IF}-\frac{f_{j}}{f_{i}-f_{j}}\cdot {\bar{N}}_{WL}\\ & =N_{NL}+B_{r,NL}-B_{NL}^{s}\\ B_{NL}^{s} & =b_{i}^{s}+\frac{cf_{2}}{\lambda_{NL}(f_{i}^{2}-f_{j}^{2})}B_{WL}^{s}\\ B_{r,NL} & =b_{r,i}+\frac{cf_{2}}{\lambda_{NL}(f_{i}^{2}-f_{j}^{2})}B_{r,WL} \end{array}\right.$$

where $N_{NL}$ denotes the integer NL ambiguity, $B_{r,NL}$ and $B_{NL}^{s}$ denote the receiver-end and satellite-end NL FCB, respectively, and c denotes the speed of light in vacuum.

Since $b^{s}$ and $d_{IF}^{s}$ are generally considered to remain relatively unchanged, $B_{WL}^{s}$ and $B_{NL}^{s}$ are also relatively stable.

### 2.2. BPNN Model

The time series of FCB often exhibit nonlinear and irregular characteristics, which cannot be accurately modeled by traditional linear methods. Among various approaches, BPNN provides a feasible pathway for constructing high-precision FCB prediction models due to its strong adaptive capability and nonlinear approximation ability.

The concept of the BPNN was first proposed in 1986. It is defined as a multiple feedforward layers neural network that is trained using the error backpropagation algorithm. Known for its simple structure, BPNN is recognized as one of the most widely used models [40].

**Figure 1:**
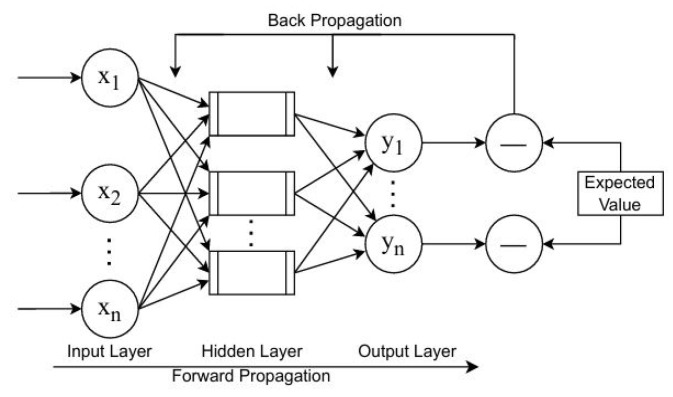
Structure for the Backpropagation Neural Network.

A standard BPNN can be divided into three parts: the input layer, the hidden layer, and the output layer. These parts are composed of some neurons, which are interconnected through weights [41], as illustrated in Figure 1.

The execution process of the BPNN consists of feedforward transmission of signals and backward transmission of errors. The general procedure is described as follows: the input data are passed through the input and hidden layers sequentially, processed layer by layer, and then delivered to the output layer. If the result does not match the expected outcome, error backpropagation is triggered. The error is propagated backward through the reverse path, and the gradient descent method is applied to update the thresholds and weights of the model, thereby reducing the error. These two steps are repeated iteratively until the prediction accuracy meets the predefined threshold condition.

In the FCB prediction model constructed in this study, the input layer consists of 14-day stable WLFCB and 5-day continuous NLFCB time series of GPS/BDS-3 satellites, while the output layer predicts 1-day WLFCB and 12 h NLFCB of the same satellites. However, FCB data do not exhibit clear patterns in their variation and distribution, making it difficult to directly establish a functional relationship between independent and dependent variables. Therefore, a sliding window method is employed to preprocess the FCB time series to construct a suitable autoregressive model. Based on the characteristics and volume of WLFCB and NLFCB data, the window sizes are 3 and 900, respectively. Thus, the autoregressive model is expressed as

(12) $$\left\{\begin{array}{l} {}[WLFCB_{m+3}]=f_{WL}(WLFCB_{m},WLFCB_{m+1},WLFCB_{m+2}),1\leq m\leq 12\\ {}[NLFCB_{n+900}]=f_{NL}(NLFCB_{n},\ldots ,NLFCB_{n+899}),1\leq n\leq 14,940 \end{array}\right.$$

where $f_{WL}$ and $f_{NL}$ represent the functional relationships corresponding to the two prediction models.

After defining the input layer, the number of neurons must also be determined, which is critical to the reliability of the BPNN. Too few neurons may lead to insufficient expressive power of the network, failing to achieve desired accuracy; too many neurons may cause overfitting and reduce computational efficiency. Therefore, selecting an appropriate number of hidden neurons is essential for reducing algorithmic complexity and ensuring good capability. However, there is no definitive approach to determine the quantity; approaches such as the trial-and-error method or Kolmogorov theorem are commonly used [42]. After comprehensive consideration of network complexity, error magnitude, and sample size, the number of hidden neurons for the WLFCB and NLFCB prediction models are 3 and 4, respectively.

### 2.3. GA-BPNN Model

The training process of a BPNN is essentially based on gradient descent to continuously adjust thresholds and weights in order to minimize the network error. The initial weights and thresholds are crucial to the performance of the BPNN. However, as they are randomly initialized prior to training, BPNN is prone to fall into local minima and converges at a slow rate, thereby compromising training efficiency.

To overcome these limitations, GA is introduced to optimize the initial parameters of the BPNN, resulting in the construction of the GA-BPNN model. The GA is an optimization algorithm inspired by biological evolutionary theory. Its core idea is to gradually improve solution quality by simulating natural processes such as inheritance, crossover, and mutation. In each generation, individuals with higher fitness are more likely to pass on their superior genes, thereby continuously enhancing the quality of the population and ultimately searching for the optimal or a near-optimal solution.

For GA-BPNN, each iteration is performed over all individuals within the population, which reduces the risk of becoming trapped in local optima. As a result, the GA-BPNN model demonstrates superior performance in FCB prediction, significantly improving both prediction efficiency and result quality.

#### 2.3.1. Basic Procedure of the GA

For the GA, the entire optimization process is composed of a series of steps, each of which determines the search efficiency and the quality of the final result. Each step is of significance and is closely interconnected with the others.

(1) Population Initialization

Before the algorithm is executed, an initial population must be defined as the starting point for evolution. The size of the initial population directly affects the convergence speed and requires a balance between search efficiency and computational complexity. In this study, the initial size is set to 5, and the maximum of generations is set to 10.

(2) Encoding

In the GA, the weights and thresholds of the BPNN must be encoded into an array in a specific order to form the chromosomes of individuals. Real number encoding is adopted in this study, with a minimum variation of 1 × 10^–6^.

(3) Fitness Evaluation

The GA aims to preserve superior individuals. To evaluate the quality of individuals and retain those with high performance, a fitness function is defined to quantify the difference between computed results and expected results. The design of the fitness function is critical for the algorithm’s ability to find the optimal solution. The fitness function defined in this study is as follows:

(13) $$F=K\cdot \sum_{k=1}^{q}\left(Q_{k}-E\right)$$

where F denotes the fitness value, $Q_{k}$ denotes the expected error, E denotes the actual error, and K denotes a constant.

(4) Selection

In the GA, the selection process identifies individuals for propagation according to their fitness levels. The roulette-wheel selection scheme is employed in this study. This method helps maintain population diversity while controlling computational cost, thereby avoiding local optima and enhancing global search capability. The selection probability of an individual is calculated as

(14) $$p(x_{i})=\frac{f(x_{i})}{\sum_{i=1}^{n}f(x_{i})}$$

where $p(x_{i})$ denotes the probability of the individual being selected, $f(x_{i})$ represents the reciprocal of the individual’s fitness, and n is the number of individuals in the population.

(5) Crossover

In the GA, the crossover operation exchanges parts of the chromosomes between two parent individuals with a specific probability to generate new chromosomes. This process aims to increase population diversity and enhance the global search capability of the algorithm. The crossover probability is set to 0.2 in this study.

(6) Mutation

In the GA, the mutation operation randomly alters part of the gene values in an individual’s chromosome with a specific probability to produce new chromosomes. This operation helps improve the local search ability and prevents premature convergence to local optima. The mutation intensity parameter is set to 2 in this study.

(7) Update

After the selection, crossover, and mutation operations, a new generation of individuals is produced. These new individuals replace part of the original population, forming an updated population. The processes of fitness evaluation, selection, crossover, and mutation are repeated until the stopping criteria are satisfied.

#### 2.3.2. Procedure of the GA-BPNN

The GA is introduced into the neural networks to optimize the initial parameters. In this way, the accuracy and efficiency of the BPNN are enhanced, providing a better starting point for subsequent training and prediction. The overall procedure of the GA-BPNN primarily consists of the following three steps:

(1) Initialization: Initial weights and thresholds are randomly generated according to the structure of the BPNN.

(2) GA optimization: The GA-BPNN model is constructed, and the initial weights and thresholds obtained in step (1) are optimized to identify a more favorable combination of initial parameters.

(3) BPNN Training and Prediction: The weights and thresholds obtained through GA optimization are assigned to the BPNN. The network is subsequently trained, and the trained model is utilized to perform FCB prediction tasks.

### 2.4. Performance Evaluation Metrics

In order to assess the training and prediction capability of the model and to directly examine the precision of the results, three metrics are employed in this study to assess the predictions, considering the inherent characteristics of WLFCB and NLFCB data.

(1) Mean Absolute Error (MAE): MAE can be used to evaluate the predictive capability of the neural network model by measuring the deviation between the true FCB and the predicted FCB. It is defined as

(15) $$MAE=\frac{1}{n}\sum_{i=1}^{n}\left|y_{i}-{\bar{y}}_{i}\right|$$

where n denotes the number of samples, $y_{i}$ represents the true FCB, and ${\bar{y}}_{i}$ represents the predicted FCB.

(2) Fractional Cycle Bias Difference (DIFF): It is used to measure the accuracy of the prediction results. By treating the FCB products provided by iGMAS as the true values, the DIFF reflects the deviation of the predicted FCB from the reference values. It is defined as

(16) $$DIFF=FCB_{t}^{s}-{\bar{FCB}}_{t}^{s}$$

where s denotes the satellite, t denotes the day of year or epoch, $FCB_{t}^{s}$ represents the FCB value provided by iGMAS, and ${\bar{FCB}}_{t}^{s}$ represents the FCB value predicted by the GA-BPNN model.

(3) Standard Deviation (STD): STD reflects the extent to which each value in a dataset deviates from the mean of the dataset. It is used to evaluate the stability of the FCB time series or the overall dispersion of the FCB difference series. It is defined as

(17) $$\sigma =\sqrt{\frac{1}{n}\sum_{i=1}^{n}{\left(y_{i}-\mu \right)}^{2}}$$

where n denotes the number of samples, $y_{i}$ represents each FCB value, and μ represents the mean of the FCB values.

## 3. Analysis of FCB Prediction Results

In this section, the stability of the preprocessed FCB time series is briefly evaluated first, followed by a comparative analysis of the prediction results between GA-BPNN and BPNN. Finally, the accuracy and quality of the predicted FCB for DOY 321 of 2023 are analyzed and validated.

The FCB products utilized in this study are sourced from iGMAS, available at the official website. The WL FCB is provided once per day, while the NL FCB product is provided every 30 s.

### 3.1. Stability Analysis of FCB Time Series

Stable, continuous, and high-precision input data are regarded as a prerequisite for accurate prediction in neural networks. The STD of the FCB time series can be utilized to represent the degree of dispersion and fluctuation range of FCB within a certain time domain, thereby intuitively reflecting the quality level of the FCB product.

#### 3.1.1. Stability Analysis of WL FCB Time Series

In this subsection, the raw WL FCB data from a 30-day period (DOY 305 to 334 in 2023) are selected for processing, and a time series stability analysis is subsequently performed. To present the results more clearly, the WL FCB values for some satellites are adjusted by ±1 cycle, confining their distribution to the range of [−1, 1.2] cycles. The addition or subtraction of one cycle does not affect the intrinsic properties of the WL FCB.

**Figure 2:**
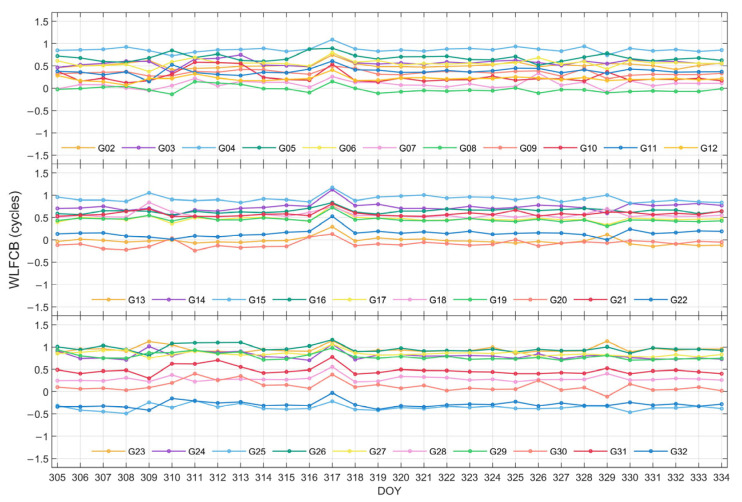
WL FCB time series of GPS with an adjustment of ±1 cycle during DOY 305–334 in 2023.

**Figure 3:**
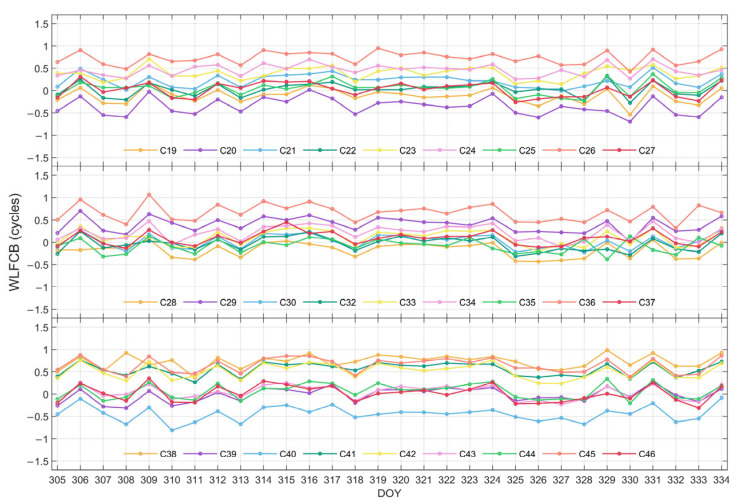
WL FCB time series of BDS-3 with an adjustment of ±1 cycle during DOY 305–334 in 2023.

The time series of WL FCB for GPS and BDS-3 satellites are plotted in Figure 2 and Figure 3, respectively. It can be observed that the WL FCB of both systems demonstrates favorable stability over the 30-day period. Among them, a jump is observed for GPS satellites on DOY 317, while for BDS-3 satellites on DOY 309 and DOY 331. Nonetheless, the WL FCB values generally stabilize before and after these jumps and remain largely stable throughout the rest of the period.

**Figure 4:**
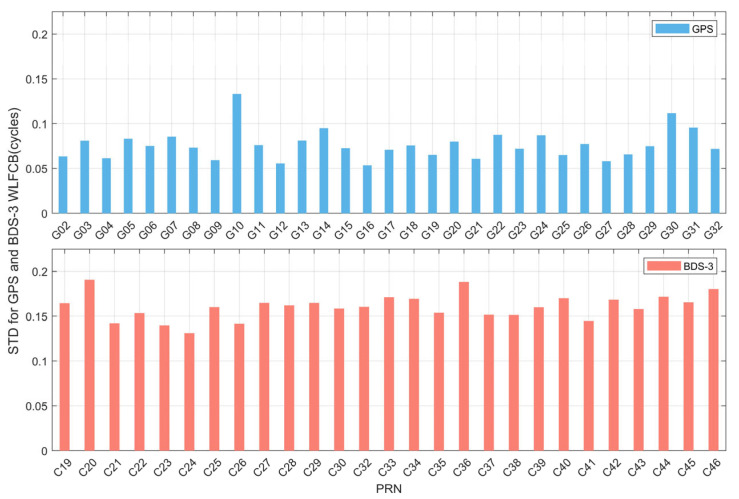
Statistical analysis of STD for GPS and BDS-3 Satellites WL FCB during DOY 305–334 in 2023.

To further analyze the quality of the WL FCB products, the STD of the WL FCB for each satellite in both systems are statistically evaluated, as illustrated in Figure 4 and Table 1. From the perspective of STD, the WL FCB STD for both satellite systems over the 30 days does not exceed 0.2 cycles. In the GPS, 93.55% of the satellites exhibit an STD below 0.1 cycles, while the STD for BDS-3 satellites is generally slightly higher, mostly concentrated within the range of 0.15 to 0.2 cycles. Furthermore, the average STD values for GPS and BDS-3 are 0.076 cycles and 0.161 cycles, respectively. It can be concluded that the WL FCB products provided by iGMAS for both GPS and BDS-3 exhibit high stability, with the variation sequences of most satellites remaining relatively stable.

#### 3.1.2. Stability Analysis of NL FCB Time Series

In this subsection, the raw NL FCB data from a 5-day period (DOY 316 to 320 in 2023) are selected for processing and analysis. Furthermore, since jumps are observed in the NL FCB across daily, the initial value of each subsequent day is aligned with the final value of the previous day during the construction of the NL FCB time series, thereby eliminating inter-day jumps.

**Figure 5:**
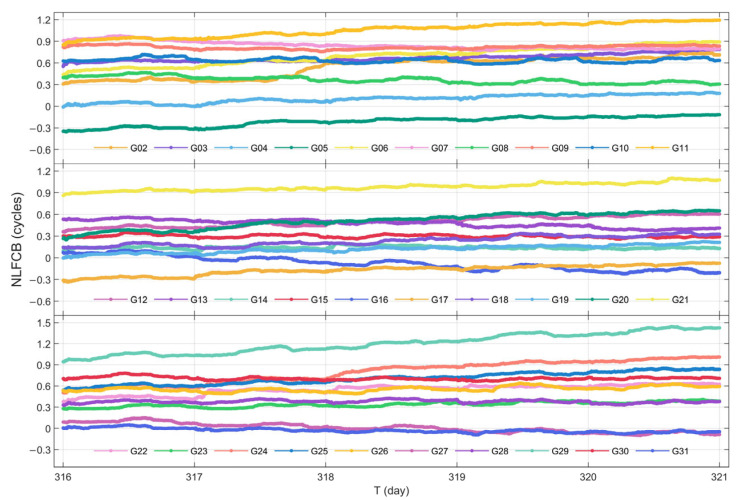
NL FCB time series of GPS during DOY 316–320 in 2023.

**Figure 6:**
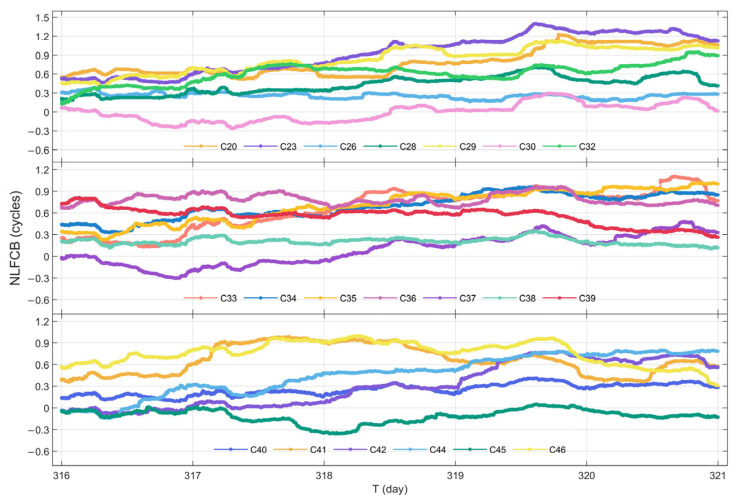
NL FCB time series of BDS-3 during DOY 316–320 in 2023.

The time series of NL FCB for GPS and BDS-3 satellites are displayed in Figure 5 and Figure 6. It can be observed that, with the exception of a few BDS-3 satellites, the majority of the satellites exhibit favorable stability and continuity in their NL FCB values. Among the BDS-3 satellites, C44 shows the largest variation in NL FCB, approaching 0.8 cycles.

To further evaluate the quality of the NL FCB products, the STD of the NL FCB for each satellite in both systems are statistically evaluated, as summarized in Table 2. From the perspective of STD, 83.87% of the GPS satellites have an STD below 0.1 cycles, while 55% of the BDS-3 satellites demonstrate an STD below 0.2 cycles. The average STD values for GPS and BDS-3 are 0.064 cycles and 0.172 cycles, respectively. It can be concluded that the NL FCB products provided by iGMAS for both GPS and BDS-3 exhibit high quality, with the variation sequences of most satellites remaining relatively stable.

Overall, the preprocessed WL FCB products and NL FCB products can be constituted into a stable, continuous, and high-precision time series, thus meeting the fundamental requirements for the input layer of neural networks and enabling high-accuracy prediction performance.

### 3.2. Comparative Analysis of BPNN and GA-BPNN

The MAE of the NL FCB sequences predicted by the BPNN and GA-BPNN models are presented in Table 3. From the perspective of the MAE metric, the performance of the GA-BPNN is significantly superior to that of the BPNN. Specifically, among the total of 50 satellites predicted, a reduction in MAE is observed for 38 satellites. Notably, the MAE of satellite G14 is reduced by 79%, representing the most prominent optimization. Overall, the average MAE values for BPNN and GA-BPNN are 0.033 cycles and 0.024 cycles, respectively, corresponding to an average improvement of 27%. This indicates that the GA-BPNN model shows better performance over the BPNN in terms of FCB prediction accuracy.

Furthermore, a comparative analysis is conducted on the number of predictions required by the two neural network models for forecasting NL FCB. As clearly demonstrated in Table 4, the number of predictions is significantly reduced by the GA-BPNN model for a considerable number of satellites, such as G24, G02, C26, and C27, with improvement rates reaching as high as 81.25%, 85.17%, 80.95%, and 93.18%, respectively. Additionally, for certain satellites (e.g., G03, G05, G06, etc.), the number of predictions remains one under both models, while a small number of satellites (e.g., G04, C41) experienced an increase in the number of predictions when the GA-BPNN is used. Overall, the average number of predictions is recorded as 4.3 for BPNN and 1.54 for GA-BPNN, indicating that the efficiency of FCB prediction is substantially enhanced by the GA-BPNN model.

In summary, based on the comparative analysis conducted in this section between GA-BPNN and the traditional BPNN, it is demonstrated that GA-BPNN exhibits superior performance. Therefore, the FCB results predicted by the GA-BPNN method are selected as the forecast product for subsequent quality analysis and PPP-AR experiments.

### 3.3. Quality Analysis of Prediction Results Based on GA-BPNN

The FCB products of DOY 321, 2023, provided by iGMAS, are selected as true values. Then, these values are processed using single difference with the FCB predictions generated by the GA-BPNN model to evaluate the deviations of the predicted values from the true values, respectively.

#### 3.3.1. Quality Analysis of WL FCB Predictions

**Figure 7:**
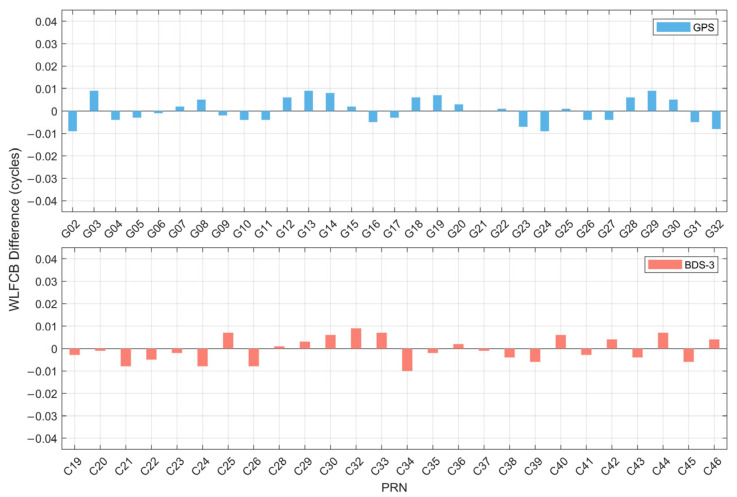
Difference between real WL FCB and predicted WL FCB of GPS and BDS-3 satellite.

Figure 7 presents the single-difference results between the predicted WL FCB and the true values for DOY 321, 2023. It can be clearly observed that the deviations between the predicted FCB and true FCB are within ±0.01 cycles for GPS and BDS-3 satellites. Specifically, among the GPS satellites, the smallest deviation is found for satellite G21, while the largest deviations (approximately 0.009 cycles) are observed for G02, G03, G24, and G29. For the BDS-3 satellites, deviations of 0.001 cycles are recorded for C20, C28, and C37, whereas the largest deviation, about 0.01 cycles, is seen in C34. Considering that the wavelength of the WL is 86.19 cm, an error of 0.01 cycles can still ensure centimeter-level accuracy for the WL.

#### 3.3.2. Quality Analysis of NL FCB Predictions

**Figure 8:**
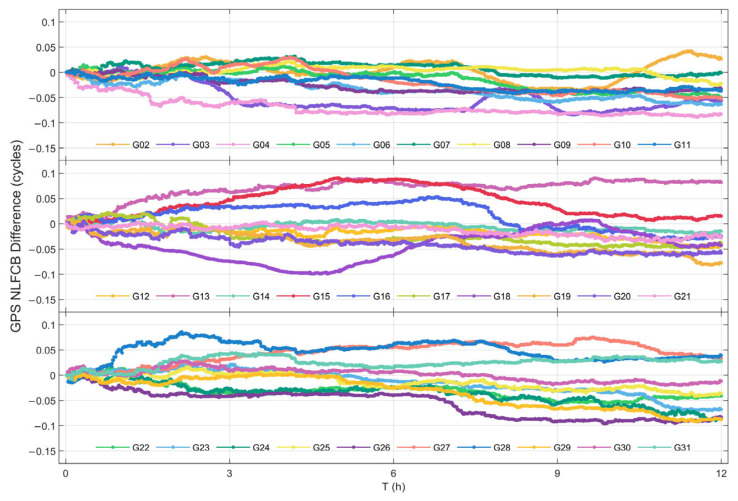
Difference between real NL FCB and predicted NL FCB of GPS satellite.

**Figure 9:**
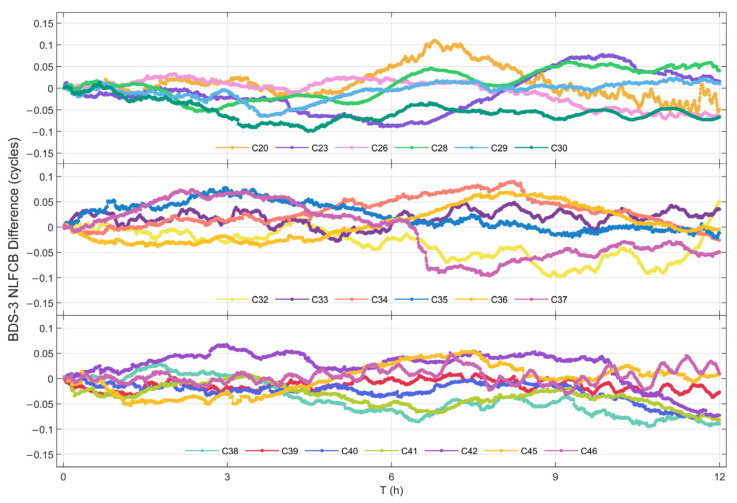
Difference between real NL FCB and NL FCB values of BDS-3 satellite.

Figure 8 and Figure 9 present the single-difference sequences between the predicted NL FCB and true values over a 12 h period for GPS and BDS-3 satellites, respectively, on DOY 321, 2023. Analysis reveals that, with the exception of satellite C20, whose deviation briefly exceeded 0.1 cycles, the deviations for all other BDS-3 satellites and all GPS satellites remained within ±0.1 cycles.

It is noteworthy that certain satellites (e.g., C20, C39) exhibited a periodic fluctuation trend during the later stage of the prediction period (approximately 9 h). This behavior is attributed to the specific construction methodology of the NL FCB prediction model. Owing to the substantial volume of NL FCB data, the model is initially constructed based on actual values at the beginning of the prediction phase. However, as the prediction progresses, previously predicted values are utilized as inputs for forecasting subsequent values. This particular model characteristic leads to the propagation and accumulation of prediction errors and data trends, thereby resulting in the observed periodic phenomena.

Table 5 statistically presents the STD and the maximum absolute deviation (MAX) of the single-difference series for NL FCB. The STD of the single-difference series reflects the overall degree of deviation for the predicted products. In general, the comprehensive deviation level of the predicted satellites is favorable, with 84% of the satellites exhibiting an STD of less than 0.03 cycles. Among these, the largest STD is observed for satellite C23, reaching 0.046 cycles. The MAX represents the deviation limit of the prediction results. Results demonstrate that only satellite C20 exceeds a maximum deviation of 0.1 cycles, while the deviations of all other satellites are controlled within 0.1 cycles. Considering that the wavelength of the NL is 10.7 cm, an error of 0.1 cycles can still ensure centimeter-level accuracy for the NL.

**Figure 10:**
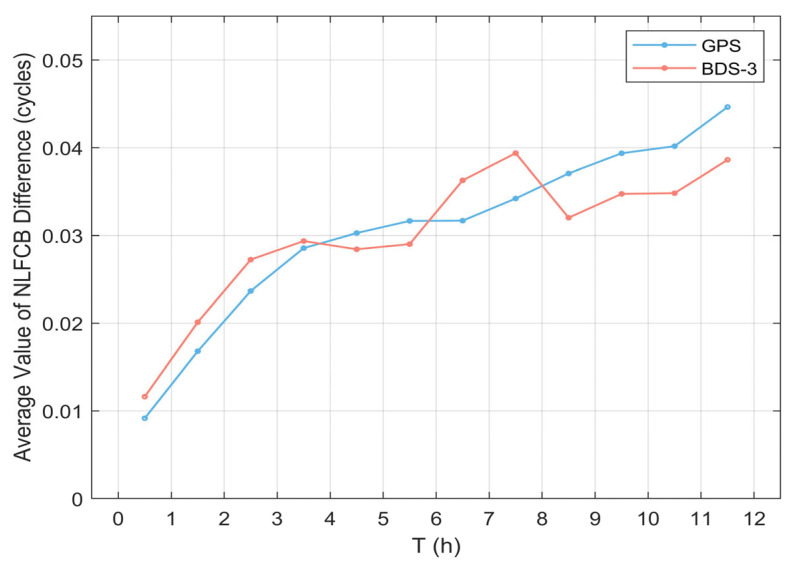
Hourly average of NLFCB difference for GPS and BDS-3 Satellite.

Figure 10 illustrates the average deviation value per hour for each satellite system, which can be used to reflect the average deviation of the prediction results on an hourly scale, thus facilitating the selection of appropriate observation periods based on the accuracy requirements of NL FCB. In general, as the prediction time increases, the single-difference sequence of the satellite NL FCB gradually diverges, and the average deviation increases overall. Specifically, the average deviation of GPS satellites during the 00:00–01:00 period is below 0.01 cycles, and the average deviation at the end of the observation does not exceed 0.05 cycles, with a steady increase over the 12 h period. For BDS-3 satellites, the average deviation during the 00:00–01:00 period is slightly higher than 0.01 cycles, but the average deviation at the end of the prediction does not exceed 0.04 cycles, with a reduction in the average deviation observed during the 08:00–09:00 period. This is because, unlike the divergence characteristic of the GPS single-difference sequence, some periodic fluctuations appear in the BDS-3 single-difference sequence, which affected the calculation of its average deviation, a result that is consistent with the findings shown in Figure 9.

In this section, the reliability and accuracy of the WL FCB and NL FCB predicted by the GA-BPNN model are evaluated. Overall, the predictive model is demonstrated to possess favorable practical utility, thereby providing theoretical support and methodological assurance for the acquisition of FCB. The following section further investigates the positioning performance of these predicted products in PPP-AR applications.

## 4. Performance Analysis of PPP-AR

**Figure 11:**
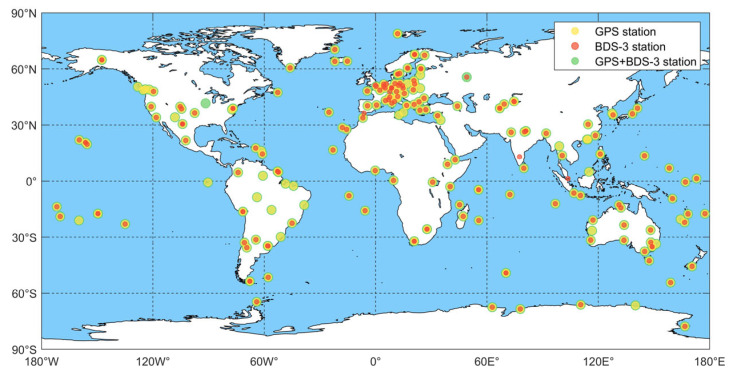
Global MGEX stations distribution used in the PPP experiments.

In this section, a substantial set of stations from the global MGEX network on DOY 321, 2023 are selected to perform positioning validation for different satellite systems. Specifically, 198 stations are used for the GPS, 176 for BDS-3, and 199 for the combined GPS + BDS-3. These stations are distributed across the region in Figure 11. Three PPP processing schemes are employed: the float solution (Float), the fixed solution based on FCB from iGMAS (Fixed), and the fixed solution based on FCB predicted by GA-BPNN (BP-Fixed). Each 12 h continuous observation file is divided into four segments, with the PPP estimation process reinitialized every 3 h. The performance of the positioning results is evaluated by analyzing the positioning accuracy, ambiguity fixed rate, and convergence time of the different PPP schemes. To assess the reliability of the results, a 95% confidence interval is applied for all statistical evaluations.

All PPP experiments are conducted using GREAT-PVT developed by the School of Geodesy and Geomatics, Wuhan University. It supports multi-GNSS PPP-AR and provides flexible configurations for different data-processing schemes, making it suitable for large-scale validation experiments such as this study [43]. Key parameter settings used for experiments are summarized in Table 6 (unlisted parameters retained their default values).

### 4.1. Positioning Accuracy Analysis

**Figure 12:**
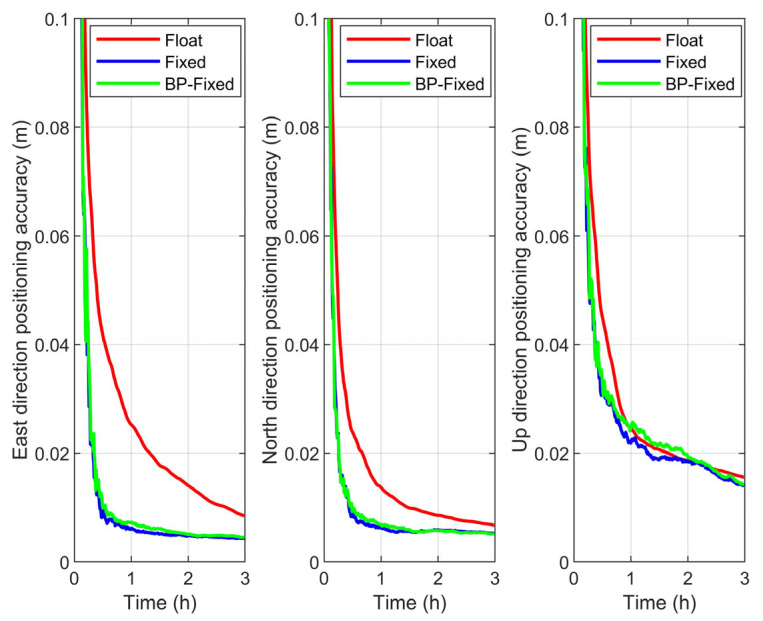
Positioning accuracy in E, N, U directions of Float, Fixed, and BP-Fixed schemes for BDS-3.

**Figure 13:**
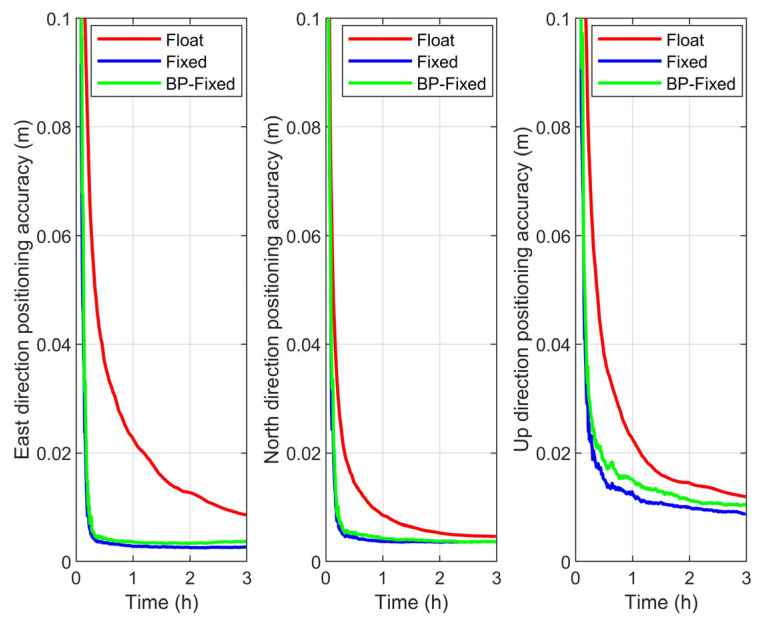
Positioning accuracy in E, N, U directions of Float, Fixed, and BP-Fixed schemes for GPS.

**Figure 14:**
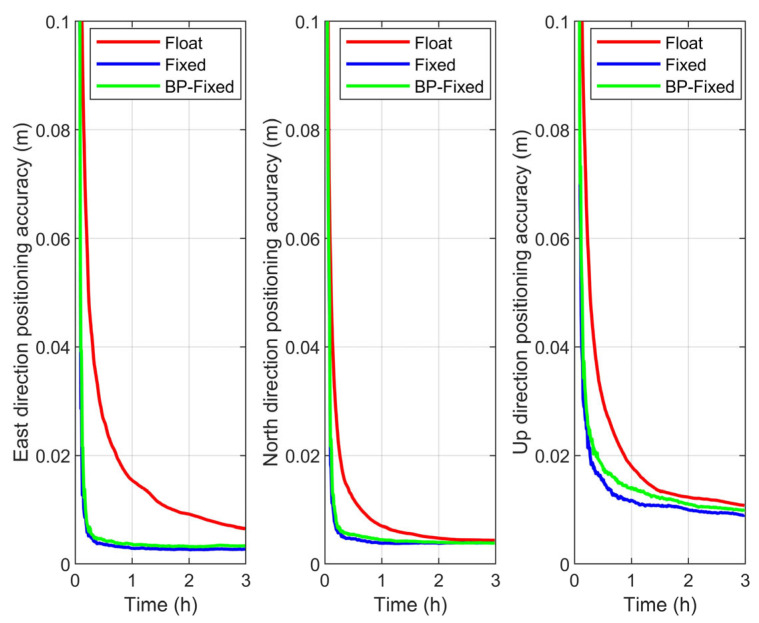
Positioning accuracy in E, N, U directions of Float, Fixed, and BP-Fixed schemes for GPS + BDS-3.

In this section, positioning accuracy is defined as the average positioning error across all observation arcs under the same observation duration. The time series of positioning accuracy for the Float, Fixed, and BP-Fixed schemes under different satellite systems are presented in Figure 12, Figure 13 and Figure 14, respectively.

From the figures, with the exception of the Up (U) direction in the BDS-3, the variation trends of the curves for all other schemes clearly reflect the improvement in convergence time achieved by the Fixed and BP-Fixed solutions compared to the Float solution. This phenomenon is most pronounced in the East (E) and North (N) directions, where the curves of the Fixed and BP-Fixed schemes for all systems almost completely coincide. This indicates that PPP-AR based on predicted FCBs achieves excellent positioning performance in the E and N directions, reaching a level comparable to that obtained using iGMAS products. In the U direction, the BP-Fixed curves for the GPS and GPS + BDS-3 schemes lie between the Fixed and Float curves, indicating that while the predicted FCBs enable ambiguity fixing in this direction, their performance has not yet reached that of the iGMAS products. Furthermore, for the BDS-3, the three curves in the U direction are relatively close to each other, suggesting that the fixing performance of both the Fixed and BP-Fixed is relatively limited in this direction.

**Figure 15:**
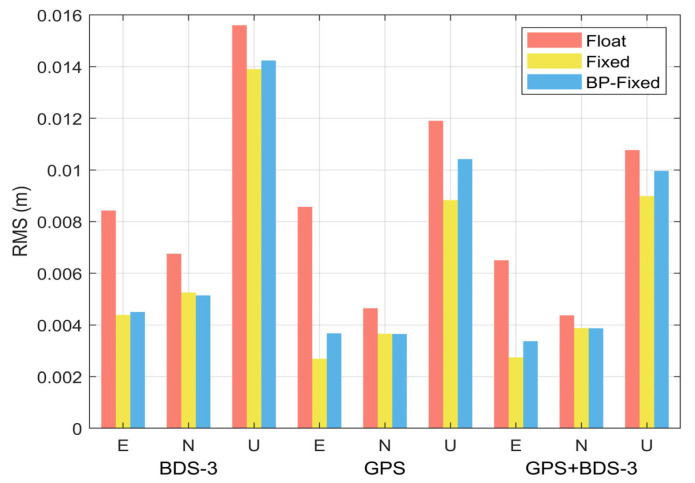
Statistical results of RMS for nine PPP schemes.

Furthermore, the value at the final epoch of the accuracy time series is defined as the RMS of the PPP scheme. The RMS of the Float, Fixed, and BP-Fixed for GPS, BDS-3, and GPS + BDS-3 are summarized in Figure 15. The following observations are clearly demonstrated by the RMS bar charts:

(1) In the E direction, regardless of the satellite constellation, the most significant improvement in accuracy is observed in both the Fixed and BP-Fixed solutions compared to the Float solution. Specifically, for BDS-3, the E-direction positioning accuracies for the Float, Fixed, and BP-Fixed schemes are 0.0084 m, 0.0044 m, and 0.0045 m, respectively. In the case of GPS, the corresponding accuracies are 0.0068 m, 0.0027 m, and 0.0037 m, whereas the combined GPS + BDS-3 achieves 0.0065 m, 0.0027 m, and 0.0034 m.

(2) For the N direction, the accuracy of the Fixed and BP-Fixed schemes is maintained at the same millimeter level. Specifically, for BDS-3, the N-direction positioning accuracies for the three schemes are 0.0068 m, 0.0053 m, and 0.0051 m, respectively. The corresponding values for GPS are 0.0046 m, 0.0037 m, and 0.0037 m, while those for GPS + BDS-3 are 0.0044 m, 0.0039 m, and 0.0039 m.

(3) For the U direction, with the exception of GPS, the positioning accuracy of the BP-Fixed scheme is notably intermediate between that of the Float and Fixed schemes, which is consistent with the conclusions drawn from the accuracy time series. Specifically for BDS-3, the U-direction positioning accuracies for the three schemes are 0.0065 m, 0.0027 m, and 0.0034 m, respectively. The corresponding accuracies are 0.0044 m, 0.0039 m, and 0.0039 m for GPS, while those are 0.0108 m, 0.0090 m, and 0.0100 m for GPS + BDS-3.

### 4.2. Analysis of Ambiguity Fixed Rate

**Figure 16:**
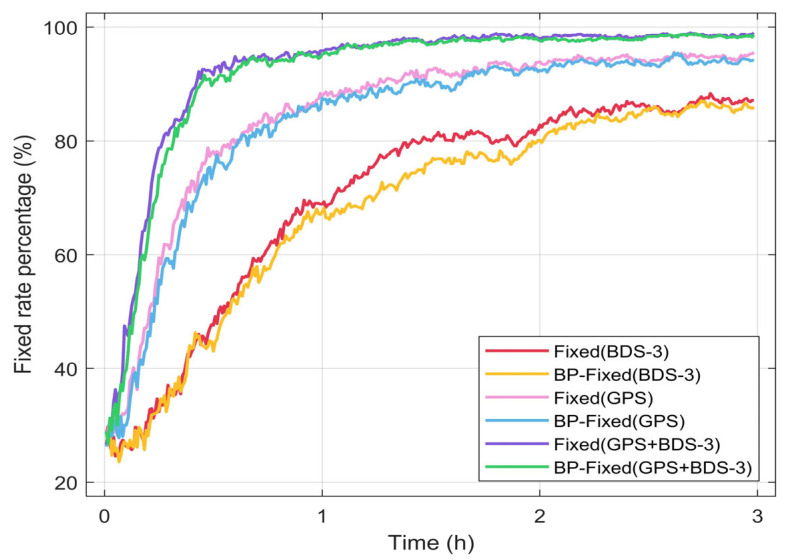
Ambiguity fixed rate for six PPP schemes.

In this section, the fixed rate is described as the statistical ratio of fixed solutions to all solutions within the same epoch. The time series of the fixed rate for the six PPP schemes are presented in Figure 16. The results indicate that the dual-system (GPS + BDS-3) achieves a notably higher fixed rate compared with either GPS or BDS-3 alone. Specifically, at the initial epoch, the fixed rate for all PPP schemes exceeds 28%. By the final epoch, the ambiguity fixed rate for GPS + BDS-3 surpasses 98%, while that for GPS remains around 95%, and BDS-3 approaches 90%.

**Figure 17:**
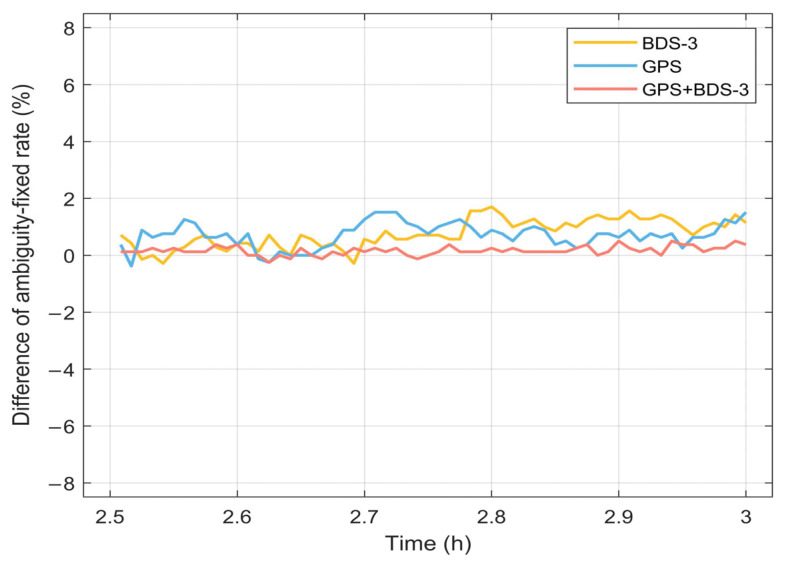
Difference in ambiguity fixed rate between Fixed and BP-Fixed schemes.

For a comparative analysis of the ambiguity fixed rates between Fixed and BP-Fixed, the rates during the last 30 min of the positioning session are selected for evaluation. The results are presented in Figure 17. It can be observed that the difference in the fixed rates under the GPS + BDS-3 scheme is the smallest, not exceeding 0.5%. The differences for the BDS-3 and GPS are also low, both remaining within 2%. The average differences in fixation rates over the 3 h period for BDS-3, GPS, and GPS + BDS-3 are 2.39%, 1.37%, and 0.90%, respectively. Therefore, in terms of the ambiguity fixed rates, the predicted FCB is demonstrated to achieve a performance comparable to that of the iGMAS products.

### 4.3. Analysis of Convergence Time

In this section, the convergence time is described as the starting time at which the three-dimensional positioning accuracy remains below 10 cm for ten continuous epochs. The convergence times for the nine PPP schemes are compared in Table 7. The convergence times of the Fixed and BP-Fixed schemes are very close, differing by no more than 0.5 min, and both are significantly lower than that of the Float scheme. Compared to the Float, the convergence time of the Fixed scheme is reduced by 7%, 14%, and 17% for BDS-3, GPS, and GPS + BDS-3, respectively, while the convergence time of the BP-Fixed scheme is reduced by 7%, 12%, and 15%, respectively. Thus, it is demonstrated that the predicted FCB can achieve convergence time comparable to that of the iGMAS products.

In this chapter, the positioning performance of three PPP schemes—Float, Fixed, and BP-Fixed—under different satellite systems (BDS-3, GPS, and GPS + BDS-3) is systematically evaluated. The results indicate that the FCB predicted by the GA-BPNN demonstrates excellent performance in multiple aspects: it effectively enables ambiguity resolution, and its performance in positioning accuracy, ambiguity fixed rate, and convergence time closely matches that of the official products.

## 5. Conclusions and Future Work

In this paper, a prediction model of FCB based on GA-BPNN is proposed, and a set of FCB products is successfully predicted, providing a new approach and methodology for acquiring high-quality FCB products efficiently.

First, the stability of the FCB products provided by iGMAS is assessed. The average values of WL FCB STD for GPS and BDS-3 over a 30-day period are 0.076 cycles and 0.161 cycles, respectively. Over a 5-day period, the average values of NL FCB STD are 0.064 cycles and 0.172 cycles.

Second, the FCB prediction results of the BPNN and GA-BPNN prediction models are compared. The mean MAE values of the two models are 0.033 cycles and 0.024 cycles, and the average number of predictions is 4.3 and 1.54, respectively.

Furthermore, by comparing with real FCB products, the reliability of the predictions is assessed from the perspective of deviation. The results show that the deviation between the predicted WL FCB and the real WL FCB is consistently controlled within 0.01 cycles. For the NL FCB, except for satellite C20, the deviations of all predicted values from the real values do not exceed 0.1 cycles.

Finally, the positioning performance of the FCB predicted products in PPP-AR is evaluated. It is demonstrated that the BP-Fixed scheme exhibits positioning performance comparable to that of the Fixed scheme. For BDS-3, compared to the Float scheme, both the Fixed and BP-Fixed schemes achieve an average improvement of 27% in positioning accuracy across the E, N, and U directions, with a 7% reduction in convergence time and a difference in no more than 2% in ambiguity fix rates during the final hour. For GPS, compared to the Float scheme, the Fixed scheme achieves an average improvement of approximately 38% across the three directions, along with a 14% reduction in convergence time. The BP-Fixed scheme attains an average improvement of about 30% and reduces the convergence time by 12%. The difference in ambiguity fix rates during the final hour between the two schemes does not exceed 2%. For the GPS + BDS-3, compared to the Float scheme, the Fixed scheme achieves an average improvement of approximately 29%, along with a 17% reduction in convergence time. The BP-Fixed scheme attains an average improvement of about 22% and reduces the convergence time by 15%. The difference in ambiguity fix rates during the final hour between the two schemes does not exceed 1%. These results prove that the PPP-AR performance using the predicted FCB achieves a level comparable to that of the products provided by iGMAS, fully verifying the feasibility and effectiveness of the proposed method in practical positioning.

In future, the application of predicted FCB products in real-time PPP ambiguity resolution will be further investigated. Efforts will be focused on improving the accuracy and efficiency of the FCB prediction model to meet the performance requirements of real-time PPP-AR.

## Figures and Tables

**Table 1:** Statistics of STD for GPS and BDS-3 WL FCB during DOY 305–334 in 2023.

Satellite System	STD Range (Cycle)	Number of Satellites	MAE (Cycle)	Average STD (Cycle)
GPS	0~0.1	29	93.55%	0.076
	0.1~0.2	9	6.45%	
BDS-3	0~0.15	5	18.52%	0.161
	0.15~0.2	22	81.48%	

**Table 2:** Statistics of STD for GPS and BDS-3 NL FCB during DOY 316–320 in 2023.

Satellite System	STD Range (Cycle)	Number of Satellites	MAE (Cycle)	Average STD (Cycle)
GPS	0~0.1	26	83.87%	0.064
	0.1~0.2	5	16.13%	
BDS-3	0~0.2	11	55.00%	0.172
	0.2~0.4	9	45.00%	

**Table 3:** Statistical of MAE of NL FCB prediction for BPNN and GA-BPNN models.

PRN	MAE (Cycle)		Improvement Rate	PRN	MAE (Cycle)		Improvement Rate
	BPNN	GA-BPNN			BPNN	GA-BPNN	
G02	0.017	0.007	59%	G27	0.045	0.023	49%
G03	0.051	0.016	69%	G28	0.049	0.056	−14%
G04	0.070	0.057	19%	G29	0.036	0.034	6%
G05	0.017	0.015	12%	G30	0.038	0.011	71%
G06	0.035	0.038	−9%	G31	0.025	0.013	48%
G07	0.012	0.019	−58%	G32	0.019	0.010	47%
G08	0.008	0.009	−13%	C20	0.054	0.036	33%
G09	0.035	0.026	26%	C23	0.047	0.046	2%
G10	0.024	0.019	21%	C26	0.039	0.029	26%
G11	0.022	0.020	9%	C28	0.032	0.033	−3%
G12	0.019	0.014	26%	C29	0.044	0.020	55%
G13	0.069	0.025	64%	C30	0.033	0.023	30%
G14	0.039	0.008	79%	C32	0.044	0.031	30%
G15	0.043	0.011	74%	C33	0.019	0.015	21%
G16	0.026	0.025	4%	C34	0.027	0.028	−4%
G17	0.029	0.021	28%	C35	0.028	0.026	7%
G18	0.045	0.025	44%	C36	0.012	0.020	−67%
G19	0.036	0.033	8%	C37	0.032	0.051	−59%
G20	0.041	0.045	−10%	C38	0.059	0.035	41%
G21	0.013	0.015	−15%	C39	0.017	0.011	35%
G22	0.032	0.013	59%	C40	0.017	0.019	−12%
G33	0.023	0.021	9%	C41	0.018	0.021	−17%
G24	0.049	0.037	24%	C42	0.034	0.033	3%
G25	0.016	0.015	6%	C45	0.029	0.021	28%
G26	0.056	0.025	55%	C46	0.020	0.015	25%

**Table 4:** Statistics of prediction attempts for BPNN and GA-BPNN models.

PRN	Count/Times		PRN	Count/Times		PRN	Count/Times	
	BPNN	GA-BPNN		BPNN	GA-BPNN		BPNN	GA-BPNN
G02	7	1	G19	3	2	C28	5	1
G03	1	1	G20	1	1	C29	17	7
G04	1	2	G21	1	1	C30	7	3
G05	1	1	G22	1	1	C32	3	2
G06	1	1	G33	1	1	C33	2	5
G07	1	1	G24	32	6	C34	3	1
G08	1	1	G25	2	1	C35	1	1
G09	1	1	G26	1	1	C36	2	1
G10	1	1	G27	2	1	C37	44	4
G11	2	1	G28	1	1	C38	1	5
G12	1	1	G29	2	1	C39	1	1
G13	1	1	G30	1	1	C40	6	1
G14	1	1	G31	1	1	C41	1	2
G15	1	1	G32	1	1	C42	13	9
G16	1	1	C20	2	3	C45	12	2
G17	2	1	C23	8	1	C46	1	2
G18	1	1	C26	21	4			

**Table 5:** Statistics of STD and MAX for NL FCB difference series.

PRN	STD (Cycle)	MAX (Cycle)	PRN	STD (Cycle)	MAX (Cycle)
G02	0.012	0.042	G27	0.026	0.075
G03	0.012	0.084	G28	0.019	0.086
G04	0.018	0.089	G29	0.032	0.092
G05	0.020	0.050	G30	0.030	0.028
G06	0.014	0.065	G31	0.009	0.044
G07	0.011	0.032	G32	0.009	0.023
G08	0.026	0.025	C20	0.036	0.110
G09	0.008	0.042	C23	0.046	0.089
G10	0.010	0.054	C26	0.029	0.071
G11	0.010	0.042	C28	0.033	0.061
G12	0.007	0.049	C29	0.020	0.065
G13	0.017	0.091	C30	0.023	0.100
G14	0.011	0.021	C32	0.031	0.098
G15	0.026	0.091	C33	0.015	0.049
G16	0.016	0.053	C34	0.028	0.090
G17	0.017	0.047	C35	0.026	0.078
G18	0.014	0.099	C36	0.012	0.069
G19	0.014	0.082	C37	0.032	0.096
G20	0.007	0.065	C38	0.035	0.095
G21	0.012	0.037	C39	0.011	0.037
G22	0.014	0.055	C40	0.019	0.082
G33	0.023	0.071	C41	0.021	0.082
G24	0.014	0.091	C42	0.033	0.075
G25	0.023	0.042	C45	0.021	0.055
G26	0.012	0.096	C46	0.015	0.051

**Table 6:** Parameter configuration.

Parameter	Configuration
Navigation File	Brdm3210.23p
Precise Orbit File	COD0MGXFIN_20233210000_01D_05M_ORB.SP3
Precise Satellite Clock Offset File	COD0MGXFIN_20233210000_01D_30S_CLK.CLK
Troposphere Model	Saastamoinen Model
Ionosphere Model	Dual-Frequency Ionosphere-Free Combination
Elevation Mask	7 deg
Positioning Mode	Static
Frequency	2
Navigation Systems	GPS/BDS-3
Ambiguity Fixed Mode	Search
Part Fix	Yes
Sampling Interval	30 s

**Table 7:** Convergence time (minutes) for nine PPP models.

Satellite System	Float	Fixed	BP-Fixed
BDS-3	19.68	18.37	18.35
GPS	16.89	14.52	14.83
GPS + BDS-3	13.81	11.42	11.77

## Data Availability

The data presented in this study are available on request from the corresponding author.
